# Shuttle Peptide Delivers Base Editor RNPs to Rhesus Monkey Airway Epithelial Cells *In Vivo*

**DOI:** 10.21203/rs.3.rs-2540755/v1

**Published:** 2023-02-17

**Authors:** Katarina Kulhankova, Soumba Traore, Xue Cheng, Hadrien Benk-Fortin, Stéphanie Hallée, Mario Harvey, Joannie Roberge, Frédéric Couture, Thomas Gross, Gregory Newby, David Liu, Alice Tarantal, David Guay, Paul McCray

**Affiliations:** University of Iowa; University of Iowa; Feldan Therapeutics; Feldan Therapeutics; Feldan Therapeutics; Feldan Therapeutics; Feldan Therapeutics; TransBIOTech; University of Iowa; Broad Institute; Broad Institute; University of California - Davis; Feldan Therapeutics; University of Iowa

**Keywords:** cystic fibrosis, adenine base editor, ribonucleoprotein

## Abstract

Gene editing strategies for cystic fibrosis are challenged by the complex barrier properties of airway epithelia. We previously reported that the amphiphilic S10 shuttle peptide non-covalently combined with CRISPR-associated (Cas) ribonucleoprotein (RNP) enabled editing of human and mouse airway epithelial cells. Here, to improve base editor RNP delivery, we optimized S10 to derive the S315 peptide. Following intratracheal aerosol of Cy5-labeled peptide cargo in rhesus macaques, we confirmed delivery throughout the respiratory tract. Subsequently, we targeted *CCR5* with co-administration of ABE8e-Cas9 RNP and S315. We achieved editing efficiencies of up to 5.3% in rhesus airway epithelia. Moreover, we documented persistence of edited epithelia for up to 12 months in mice. Finally, delivery of ABE8e-Cas9 targeting the *CFTR R553X* mutation restored anion channel function in cultured human airway epithelial cells. These results demonstrate the therapeutic potential of base editor delivery with S315 to functionally correct the *CFTR R553X* mutation in respiratory epithelia.

## Introduction

Gene editing offers the opportunity to repair or modify mutations associated with inherited diseases such as cystic fibrosis (CF), a disorder caused by mutations in cystic fibrosis transmembrane conductance regulator (*CFTR*). While *CFTR* small molecule modulator therapies are available for certain mutations, the products of *CFTR* premature termination codon (PTC) alleles are not responsive. Base editing using adenine deaminase proteins provides an opportunity to repair PTCs by efficiently installing single base changes with minimal undesired byproducts.

Adenine base editors (ABEs) employ catalytically inactive or nickase Cas variants fused to an evolved deoxyadenosine deaminase protein to specifically target point mutations by converting an A•T to G•C^1^.Notably, these RNA-guided programmable editors catalyze site specific single nucleotide conversions without DNA double-strand breaks. Base editing relies on ubiquitously expressed cellular mismatch repair machinery and does not require cell division^2^. Thus, the base editing process may proceed efficiently in post-mitotic cells *in vivo*, overcoming a limitation in the airways for strategies requiring homologous recombination^3, 4^.

For disorders involving the respiratory tract such as CF, a critical challenge is the delivery of the gene editing payload to the epithelial cells of the conducting airways. We and others recently demonstrated the feasibility of using ABEs to correct *CFTR* PTC mutations when delivered to cultured airway epithelial cells as RNPs via electroporation^5^ or as mRNA^6^. Several delivery strategies including viral and non-viral vectors are in development to enable the delivery of editing reagents to somatic cells *in vivo*^7, 8, 9, 10, 11^. One challenging but attractive approach is to deliver ABEs as an RNP complex. In contrast to coding RNA or DNA, RNP delivery allows immediate and efficient editing while the transient cell exposure limits the off-target opportunities. While Yeh et al. successfully delivered Cas9-BE3 (CBE) RNPs to edit inner ear cells of mice using cationic lipids^2^, we are unaware of an effective lipid-based reagent for RNP delivery to airway epithelia^12^. We previously reported successful CRISPR-associated nuclease delivery to the respiratory tract of mice using an engineered amphiphilic S10 shuttle peptide^12^. We achieved editing of loxP sites in airway epithelia of ROSA^mT/mG^ mice, offering potential avenues for Cas and base editor RNP delivery to refractory airway epithelial cells *in vivo*.

Here, we identified an optimized shuttle peptide and prepared recombinant ABE8e-Cas9 RNP to investigate shuttle-mediated delivery and base editing efficiency in relevant airway epithelial cells. To our knowledge, we demonstrate for the first time the feasibility of base editing of airway epithelia in the rhesus monkey model. In addition, using the Ai9 ROSA26 tdTomato reporter mouse model we document the persistence of *in vivo* edited airway epithelial cells for 12 months. Importantly, the efficiency of editing with this delivery strategy was sufficient to restore *CFTR* function in cultured primary CF airway epithelial cells.

## Results

**Figure 1:**
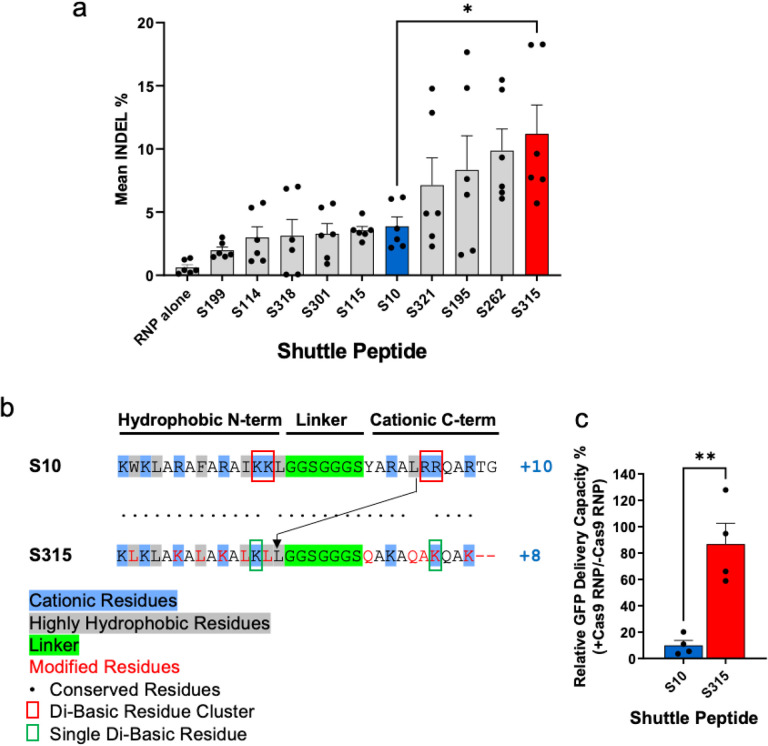
Identification of shuttle peptides with improved delivery of Cas9 RNPs to airway epithelia. **a.** Delivery of Cas9 RNP targeting *CFTR* locus in human airway epithelial cells cultured at the air liquid interface using indicated shuttle peptide candidates. Y axis represents the frequency of InDels attained with the indicated peptide. Individual closed circles represent averaged data from an individual donor. Results plotted as mean ± SEM, * P<0.05. **b.** Comparison of the amino acid sequences of S10 and S315 peptides. **c.** Inhibitory effect of Cas9 RNP on S10- or S315-mediated delivery of GFP to CFF-16HBEge cells. GFP (10 μM), S10 or S315 (10 μM) peptide with or without Cas9 RNP (containing 2.5 μM Cas9 and 2 μM gRNA) were added to cells and GFP delivery quantified by flow cytometry. The Y axis represents the relative delivery activity (%), calculated as the GFP delivery attained with or without Cas9 RNP addition. Results plotted as mean ± SEM, ** P<0.005. Individual circles represent data from biological replicates.

Identification of a shuttle peptide with improved delivery of ABE8e-Cas9 RNPs to airway epithelia. We previously demonstrated the successful delivery of green fluorescent protein (GFP) and Cas12a and Cas9 RNPs to airway epithelia *in vitro* and *in vivo* using the amphiphilic S10 peptide^12^. From a peptide screen in well differentiated primary cultures of human airway epithelia grown at an air-liquid interface we identified four new peptides (termed S321, S195, S262, and S315) that delivered Cas9 RNP more efficiently than S10 (Fig. 1a and Supplementary Table 1). Based on this result, we asked whether the improved Cas9 RNP delivery to human cells by S315 could be adopted for gene editing in airway epithelia.

The primary amino acid sequences of the S315 peptide were derived from the S10 sequence and features a similar N-terminal hydrophobic cluster and C-terminal hydrophilic/cationic tail, and an identical poly-glycine linker (Fig. 1b). Note that the S315 peptide has a lower cationic charge density (+8) than S10 (+10). Both di-basic residue clusters found in S10 (KK/RR highlighted by red squares) were each reduced to a single residue in the S315 peptide (green squares). To reduce the hydrophobicity of the C-terminus, the single leucine in the S10 C-terminus was moved to the N-terminus of S315 (arrow). Finally, for uniformity, the S315 peptide contains only leucines (L) as highly hydrophobic residues, lysines (K) as di-basic residues, and glutamines (Q) or alanines (A) as uncharged residues.

We previously found that S10-mediated delivery of Cas12a RNP with its shorter gRNA was more efficient than S10 delivery of Cas9 RNP. This may be due to inhibitory effects that the greater anionic charge density of the longer Cas9 gRNA has on peptide-mediated delivery^12^. As S315 presents a lower overall charge (+8) than S10 (+10) and lacks both KK and RR cationic clusters, we investigated the sensitivity of S10 and S315 peptides to inhibition of delivery by the polyanionic charges associated with gRNAs using Cas9 RNPs (Fig. 1c and Supplementary Fig. 1). To determine the relative activity maintained in the presence of Cas9 RNP, we applied GFP to the CFF-16HBEge human bronchial epithelial cell line in the presence or absence of Cas9 RNPs and quantified GFP delivery by S10 using flow cytometry as previously described^12^. The inclusion of Cas9 RNPs markedly reduced the GFP delivery efficiency using S10, while addition of Cas9 RNPs had little impact on S315 peptide-mediated delivery which retained ~ 85% of its delivery activity. These results suggest that the improved Cas9 RNP delivery by S315 is at least partially due to a reduced susceptibility to inhibition by the Cas9 RNP.

**Figure 2:**
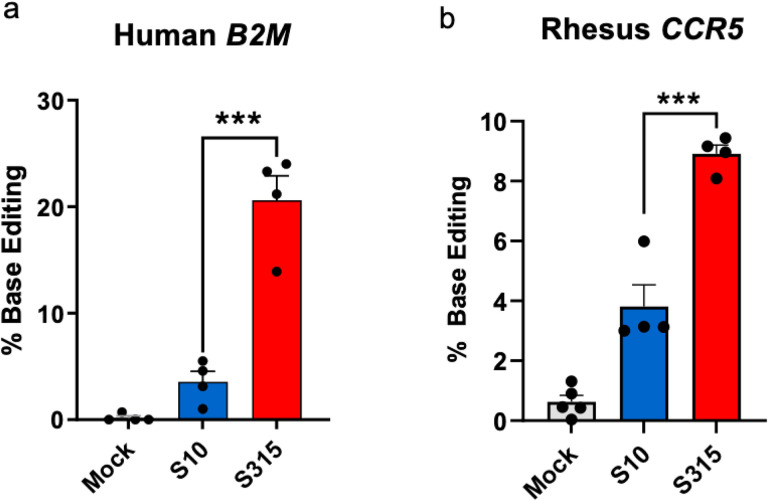
Adenine base editing in human and rhesus monkey airway cells in vitro. **a.** Delivery of ABE8e-Cas9 RNP targeting *B2M*locus using S10 and S315 peptides in human airway epithelial cells cultured at the air liquid interface. Editing efficiency was assessed using HTS. Individual closed circles represent data from technical replicates. *** P< 0.001. **b.** Delivery of ABE8e-Cas9 RNP targeting *CCR5* locus using S10 and S315 peptides in rhesus tracheal epithelial cells cultured at the air liquid interface. Editing efficiency quantified using HTS. Individual closed circles represent data from technical replicates. *** P< 0.001.

Adenine base editing in human and rhesus monkey airway cells in vitro. As Cas9 and ABE8e-Cas9 share the same sgRNA, we next compared the utility of S10 and the more efficient S315 peptide for ABE8e-Cas9 RNP delivery to the CFF-16HBEge cells grown in submersion culture^13^. The frequency of ABE8e-Cas9 base editing at the targeted B2M locus was quantified by high-throughput DNA sequencing (HTS). The editing efficiency for ABE8e-Cas9 RNP delivered by the S315 peptide was ~ 20%, a significant increase over the S10 peptide (Fig. 2a). To investigate the feasibility of adenine base editing in the rhesus monkey model, we targeted the safe harbor *CCR5* locus, which we could assess *in vivo* in future experiments. We delivered ABE8e-Cas9 RNPs targeting the *CCR5* locus to primary cultures of rhesus monkey tracheal epithelial cells grown at an air-liquid interface using the S10 or S315 peptide. As shown in Fig. 2b, the editing attained using the S315 peptide was greater than S10, achieving a mean editing efficiency of ~ 9%.

Amphiphilic peptides enhance delivery of a protein cargo to airway epithelia in vivo. To evaluate shuttle peptide delivery biodistribution in vivo, we designed a nuclear targeted peptide cargo labeled with a Cy5 dye. To generate this cargo, we synthesized a peptide comprised of a nuclear localization signal (NLS). We used D-amino acids to avoid protease degradation and a retro-inverso sequence to conserve the amino acid residue disposition of the NLS^14^. Finally, we chemically conjugated a sulfo-Cy5 fluorophore to a cysteine residue at the peptide C-terminal end to generate the NLS-Cy5 cargo as described in Methods. When co-administered with a shuttle peptide, the nuclear Cy5 signal signifies successful delivery and avoidance of the endosomal entrapment often associated with cell penetrating peptides (CPP). In these experiments we used the S10 peptide since its GFP delivery activity in absence of Cas9 RNP was greater than that observed with S315 (Supplementary Fig. 1). As outlined in the Methods, we delivered 1 ml of NLS-Cy5 (10 μM) formulated with the S10 peptide (40 μM) into the trachea of young rhesus monkeys using an atomizer. Between one and two hours post-delivery animals were euthanized, and lung lobes collected, fixed, and prepared for examination (Supplementary Table 2, Group 1).

**Figure 3:**
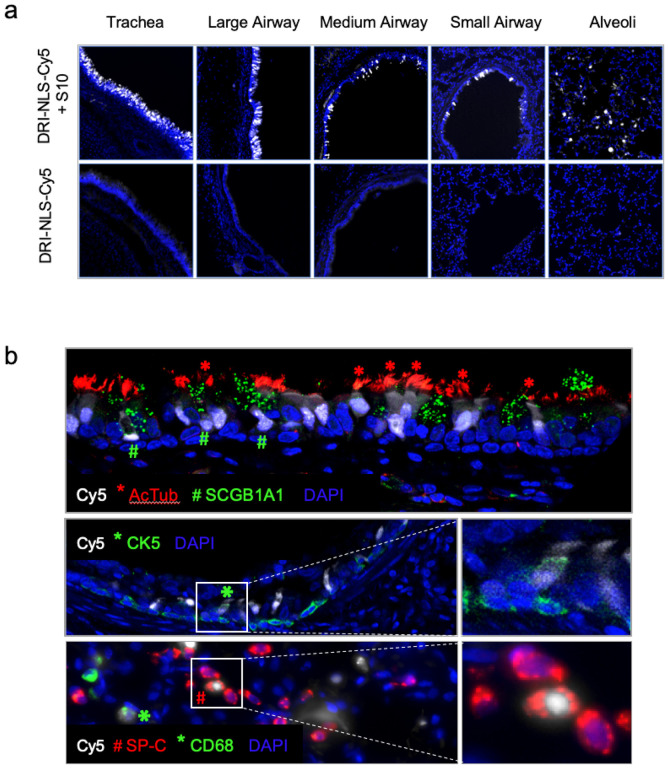
Intratracheal *in vivo* delivery of DRI-NLS-Cy5 with S10 shuttle peptide results in widespread distribution and cell-type specific delivery in the rhesus monkey airways. **a.** Representative epifluorescence microscopy images of the rhesus lung tissue sections demonstrating the DRI-NLS-Cy5 localization in the surface epithelial cells of airways of various sizes, ranging from trachea to small airways and alveolar regions (Fig. 3a, upper panels). Control received DRI-NLS-Cy5 alone (Fig. 3a, lower panels). **b.** Confocal microscopy image documenting localization of DRI-NLS-Cy5 fluorescence (white) in ciliated cells (*, AcTub - red), secretory cells (#, SCGB1A1 - green) – top panel, and very rarely to CK5^+^ basal cells (*, CK5 – green), middle panel, where DAPI is pseudo-colored blue. In the alveolar regions (Fig. 3b, lower panel), the DRI-NLS-Cy5 signal (white) localized to the nuclei of the surfactant protein C producing alveolar type II cells (#, SP-C - red) and alveolar macrophages (*, CD68-green).

Using epifluorescence (Fig. 3a) and confocal microscopy (Fig. 3b) we documented NLS-Cy5 signal in surface epithelial cells of the large and small conducting airways, and the alveolar regions. We observed Cy5 signal in ciliated cells (acetylated tubulin^+^) and secretory cells (SCGB1A1^+^) of the large cartilaginous airways and in smaller airways lined by a cuboidal epithelium (Fig. 3b). To assess delivery to cells with progenitor capacity we focused on airway basal cells. In the pseudostratified columnar epithelium of the conducting airways the basal progenitor cells are located below the differentiated surface cell types. In the small airways typified by a cuboidal epithelium, the basal progenitor cells have an apical membrane that is accessible from the lumen^15^. Sporadic co-localization of Cy5-NLS with the basal cell marker cytokeratin 5 (CK5) was observed (Fig. 3b).

**Figure 4:**
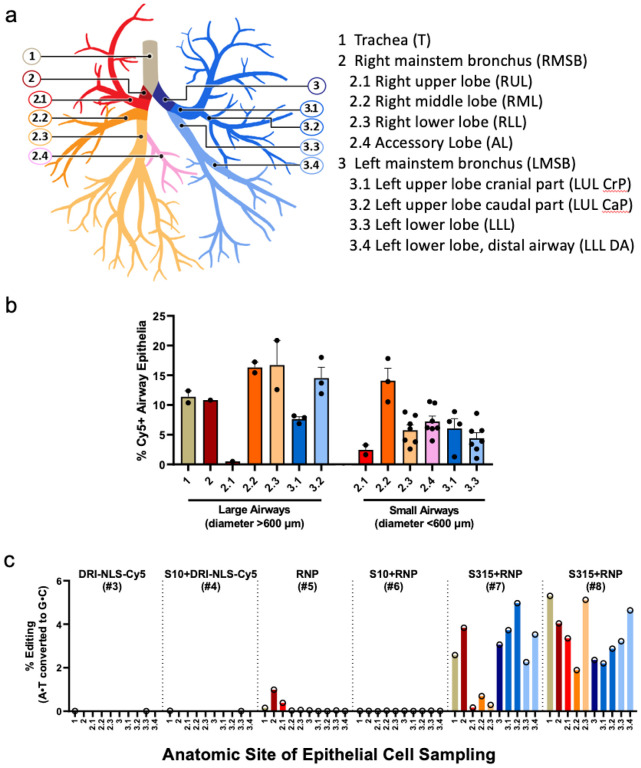
Quantification of *in vivo* delivery of DRI-NLS-Cy5 and ABE8e-Cas9 RNP to rhesus respiratory epithelia. **a.** Diagram of rhesus monkey airway tree. The regions where airway tissue sections or cytology brushings were obtained are color coded and numbered as indicated. **b.** Quantification of DRI-NLS-Cy5 delivery with S10 peptide in trachea (T), right mainstem bronchus (RMSB), and 7 lobes (RUL - right upper lobe, RML - right middle lobe, RLL - right lower lobe, AL - accessory lobe, LUL-CrP - left upper lobe, cranial part, LUL CaP - left upper lobe, caudal part, LLL - left lower lobe). Each circle represents one airway analyzed in a single tissue section, and columns represent mean ± SEM. **c.** Efficiency of shuttle peptide mediated Cas9-ABE8e RNP editing at *CCR5* locus scored by airway region. Y axis indicates A to G editing efficiency. X axis denotes conditions including DRI-NLS-Cy5 alone (#3), S10+DRI-NLS-Cy5 (#4), ABE8e-Cas9 RNP alone (#5), S10+ABE8e-Cas9 RNP (#6), and S315+ABE8e-Cas9 RNP (#7, 8). Each condition presents data from an individual animal. The animal numbers correspond to conditions described in Supplementary Table 2. n=6 animals.

In the lung parenchyma, we identified cells with NLS-Cy5 positive nuclei that co-localized with surfactant protein C (SP-C), a marker of alveolar type II cells (Fig. 3b). Alveolar type II cells are a progenitor cell in the lung parenchyma. Some NLS-Cy5 positive cells were also observed in the alveolar lumen that co-localized with CD68, a marker for alveolar macrophages. In macrophages the Cy5 signal was cytoplasmic, suggesting ingestion of free NLS-Cy5 peptide, rather than transduction via shuttle peptide delivery (Fig. 3b). This result provides further support that shuttle peptides designed to specifically deliver proteins to the cell cytoplasm facilitate efficient import of the NLS-tagged cargo to the nuclei of airway epithelial cells. The observed heterogeneity in the percentage of cells targeted regionally presumably reflects variable delivery of materials from a single intratracheal aerosol bolus. Figure 4a shows a schematic of rhesus airway anatomy. Figure 4b presents the regional distribution of the NLS-Cy5 peptide signal in trachea, bronchus, and large and small airways of the five lung lobes sampled; the bars are color coded to correspond with Fig. 4a. The range of NLS-Cy5 labelled cells was 0.5–20.8% in the large airways and 1% − 17.8% in the small airways.

Successful A > G editing of the *CCR5* locus in rhesus monkey airway epithelia *in vivo*. Using an approach identical to that outlined for the Cy5-NLS peptide, we delivered ABE8e-Cas9 RNP (2.5 μM Cas/2 μM gRNA) formulated with either the S10 or S315 peptide (40 μM) by intratracheal aerosol as described in Methods. All animals were screened for study selection and those negative for antibodies to SpCas9 assigned to the study. Seven days later, animals were euthanized, lung lobes collected, airway epithelia obtained using cytology brushes, and DNA extracted and subjected to HTS (Supplementary Table 2, Group 2).

The airway regions sampled by cytology brushings are shown schematically in Fig. 4a. The editing efficiencies in the trachea, left and right mainstem bronchi, and segmental bronchi are presented in Fig. 4c. Controls included animals that received Cy5-NLS alone, Cy5-NLS + S10 shuttle, and ABE8e-Cas9 RNP with no shuttle (Supplementary Table 2, animals 1–4, 5). Little editing was observed at the *CCR5* locus with the ABE8e-Cas9 RNP alone or S10 peptide delivery of the ABE8e-Cas9. In contrast, in the two animals that received the S315 shuttle peptide with ABE8e-Cas9 RNP we measured a mean base editing efficiency of 2.8% (range 0.2–5.3%) depending on the airway region sampled (Fig. 4c). The most efficient editing was observed in the left upper lobe caudal part (5%), left upper lobe cranial part (3.7%), and the right mainstem bronchus (3.8%) in monkey #7 and in the trachea (5.3%), right lower lobe (5.1%) and left lower lobe (4.6%) in monkey #8, both which received the S315 shuttle peptide.

**Figure 5:**
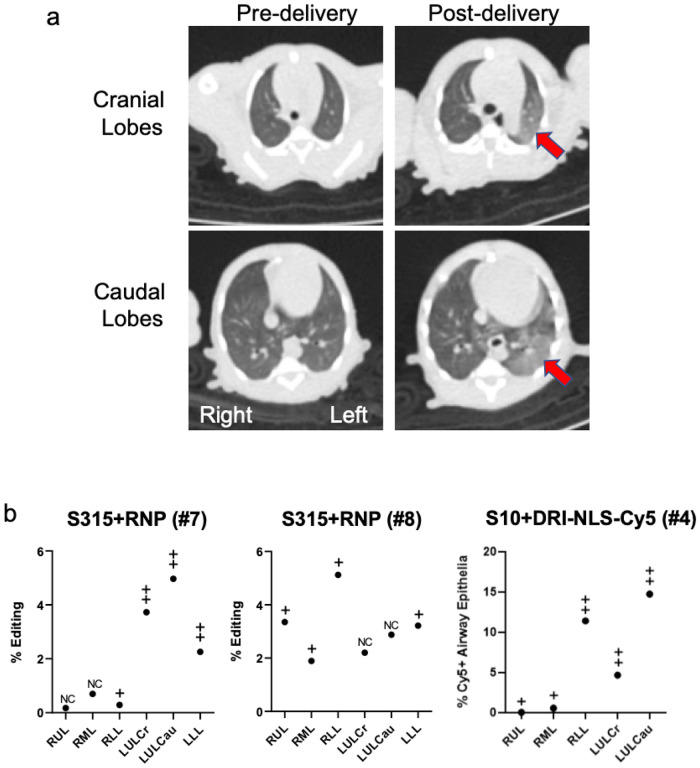
Application of chest CT scans to identify regions of deposited base editing reagents. **a.** Chest CT images from monkey #7 (S315+RNP) from panel (b) below. Arrows highlight areas of consolidation. **b.** Correlation between regional editing efficiency or regional DRI-NLS-Cy5 nuclear localization and areas of ground glass opacity or consolidation on CT scan. Regions studied include RUL - right upper lobe, RML - right middle lobe, RLL - right lower lobe, LUL-Cr - left upper lobe, cranial part, LUL Ca - left upper lobe, caudal part, LLL - left lower lobe. The CT scans were scored in a blinded fashion for changes in aeration as follows: NC: no change from baseline; + subtle nonsegmental ground glass; ++ segmental ground glass; +++ dense consolidation. Filled circles represent editing efficiencies for indicated region as shown in Fig. 4c.

We obtained CT scans in all animals pre- and immediately post-intratracheal aerosol delivery of all cargoes. We anticipated heterogenous deposition of editing materials within the airway tree following a single 1 ml aerosol administration. As an example, Fig. 5a displays the pre- and post-delivery chest CT images from monkey #7 (S315 + RNP). We hypothesized that changes in aeration post-delivery might serve as a surrogate for regions receiving the greatest aerosol deposition. The CT scans were scored for areas of ground glass changes or consolidation post-procedure by a pulmonologist blinded to the experimental conditions. CT scans were examined in multiple planar reconstructions and the parenchymal abnormalities then assigned to corresponding proximal conducting airways per the anatomic schema in Fig. 4a. A four-grade scale (ranging from no change (NC) to +++) was used to characterize new findings in individual lung lobes after cargo delivery. The results are presented in Fig. 5b. Indeed, the areas with the greatest CT scan changes characterized and scored as nonsegmental or segmental ground glass changes correlated with greater *CCR5* locus editing (monkeys #7, 8) or Cy5 + cells (monkey #4), suggesting greater reagent deposition in those regions. Clinical serum chemistries and complete blood counts (CBCs) obtained immediately pre-delivery and at time of euthanasia were all within normal limits between control, Cy5-NLS, and ABE8e-Cas9 treated animals.

**Figure 6:**
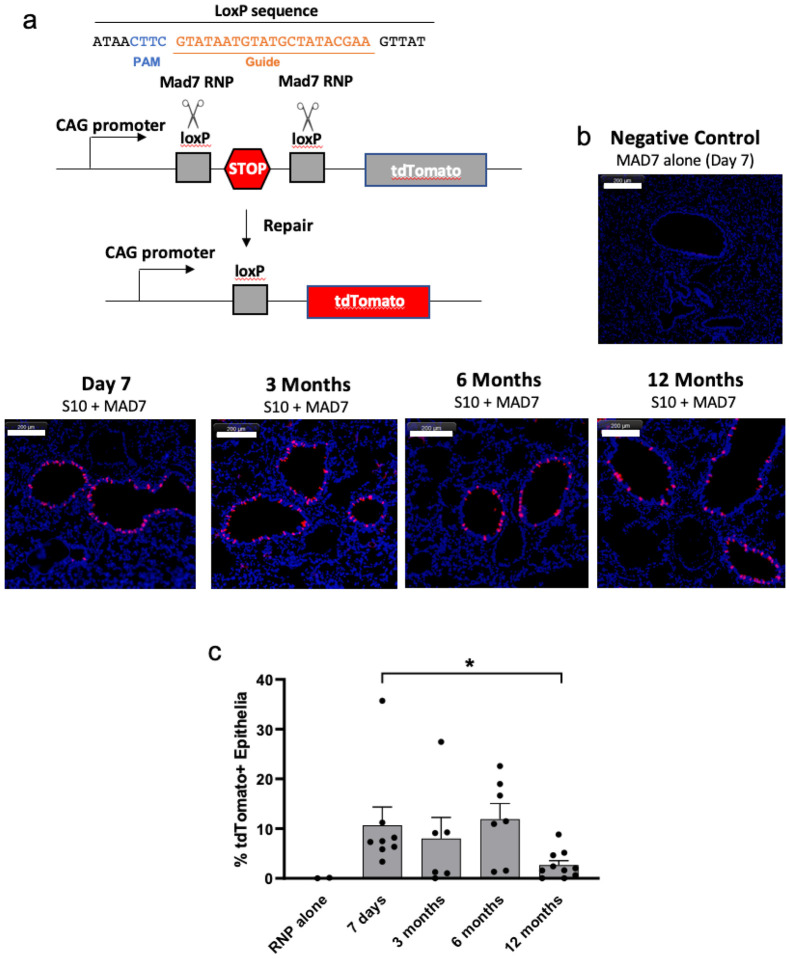
Persistence of gene editing in Ai9 mice following S10-mediated delivery of MAD7 nuclease. **a.**Schematic of reporter in Rosa26 locus of Ai9 mice. PAM and gRNA to target LoxP sites are shown. **b.** Ai9 mice received MAD7 RNP with the S10 shuttle as described in the Methods section. At the indicated intervals, editing was assessed in lung tissue sections using fluorescence microscopy. Nuclease dependent editing is signified by tdTomato expression. White scale bar indicates 200 μm. **c.** Quantification of tdTomato expression in airway epithelia at the indicated intervals. Each dot represents the counting of an individual mouse and columns represent ±SEM, *denotes P<0.05 by Kruskal-Wallis test.

In Vivo persistence of editing. Our data demonstrate that shuttle peptide delivery mainly targets the accessible surface ciliated and secretory cells in the airways (Fig. 3). To investigate the long term persistence of gene edited airway epithelial cells *in vivo*, we used the Ai9 ROSA26 tdTomato mouse model^16^. The main surface epithelial cell types of the murine airways are ciliated and secretory. In these animals, nuclease mediated excision of a LoxP flanked stop codon in the *Rosa*26 locus activates tdTomato expression (Fig. 6a). Using methods similar to a previous report^12^, the MAD7 nuclease^17^ RNP was delivered intranasally using the S10 peptide. MAD7 is an engineered Cas12a variant type isolated from the bacterium *Eubacterium rectale*. At intervals of 1 week and 3, 6, and 12 months lung tissues were collected and tdTomato expression in epithelial cells evaluated and quantified using fluorescence microscopy as described in Methods (Fig. 6b). We observed persistence of tdTomato expressing cells over 12 months with an average of ~ 8.3% edited airway epithelial cells across all time points (Fig. 6c). There was a decline in the number of tdTomato^+^ cells from 7 days to 12 months.

**Figure 7:**
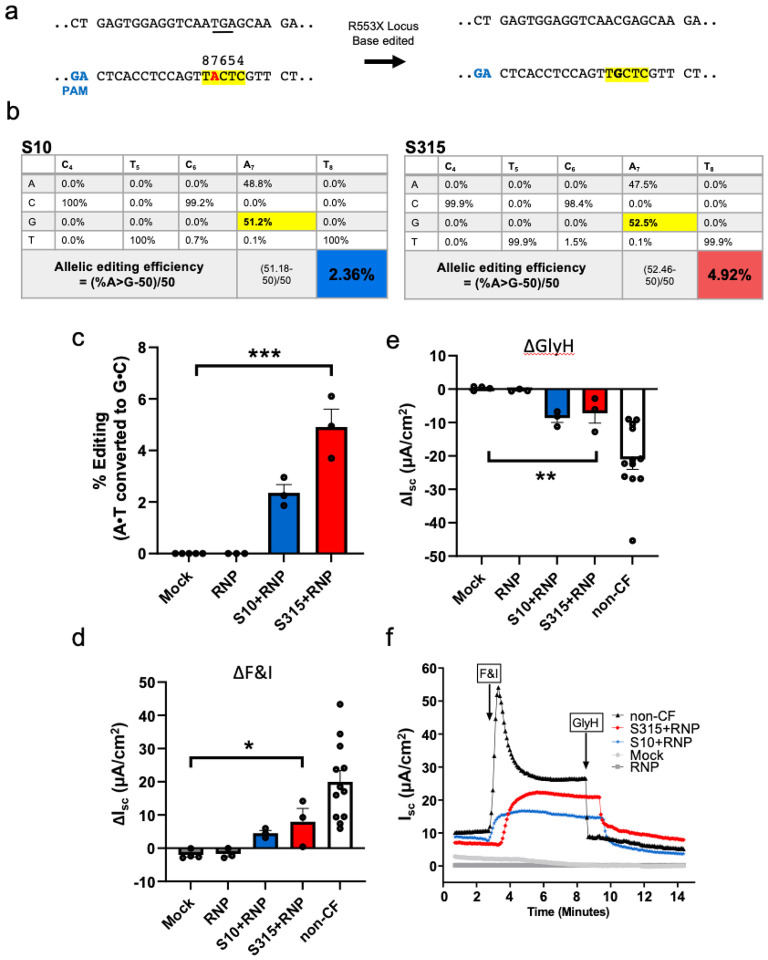
Shuttle peptide delivery of ABE8e-Cas9 RNP to primary air liquid interface cultures of human airway epithelial cells (*R553X*/L671X) targeting *R553X*locus. One week following the first application of ABE8e-Cas9 RNP delivery with shuttle peptides, Ussing chamber analysis was conducted, and DNA editing was analyzed by HTS. **a.** Top panel show the target DNA strands and PAM sites (blue text). Yellow highlight denotes the predicted 4–8 nt ABE editing window (numbered). Mutations highlighted in red text. **b.** Average frequency of desired product and allelic editing efficiencies for *R553X* nonsense mutation with the indicated shuttle peptides. Percent allelic editing efficiencies calculated by (% base edited-50)/50 *100) and graphically represented in panel (**c**). Statistics by one-way ANOVA, ***P<0.001. **d, e.** *CFTR*-dependent anion channel activity summarized from short circuit current tracings across all treatment groups. Change in short circuit current (ΔIsc) in response to F&I (d) and GlyH (e) in groups represented in (c) and non-CF donor cells. Statistics by one-way ANOVA, *P<0.05, **P<0.005. **f.** Representative short circuit current tracings comparing mock, ABE8e-Cas9 RNP alone ABE8e-Cas9 RNP + S10, and ABE8e-Cas9 RNP + S315 treated cells. Three technical replicates for each treatment group. Each data point represents one culture. (n=3 technical replicates).

ABE8e-Cas9 RNP delivery to CF airway epithelia partially restores *CFTR* anion channel function. We next asked if ABE8e-Cas9 RNP delivery with the S315 peptide could restore function to cells with a *CFTR* nonsense mutation. Since there were no rhesus monkey models of CF available, we performed these studies in primary human airway epithelial cells heterozygous for the mutation *R553X* mutation (*R553X*/L671X). We produced and purified a ABE8e-Cas9 NG protein for this study as no NGG PAMs are available at the human *R553X* locus (Fig. 7a). ABE8e-Cas9 RNP was delivered to well differentiated air-liquid interface cultures as described in Methods^18^. One week following ABE8e-Cas9 delivery, editing was quantified by HTS and *CFTR*-dependent Cl^−^ secretion was measured. The editing efficiency achieved with S315 delivery (4.91%) exceeded that observed with the S10 peptide (2.36%) (Fig. 7b, c). To evaluate the functional impact of the A•T to G•C editing, we measured CFTR-dependent anion channel activity in Ussing chambers. Epithelia were sequentially treated with amiloride to inhibit epithelial sodium channels (ENaC) and DIDS to inhibit non-CFTR Cl^–^ channels. We next applied forskolin and IBMX (F&I, cAMP agonists) to activate CFTR-dependent Cl^–^ secretion (measured as change in short circuit current, Δ/_sc_, Fig. 7d). Activation of *CFTR* was assessed by the addition of the CFTR channel inhibitor GlyH-101 (GlyH, Fig. 7e). We observed significant increases in CFTR-dependent Cl^–^ transport following ABE8e-Cas9 delivery with S10 and S315 peptides (Fig. 7d, e). Representative tracings from these experiments are shown in Fig. 7f. The CFTR-dependent short circuit current observed following S315 mediated delivery was greater than that of S10, consistent with the HTS results.

## Discussion

Here we report the first demonstration of the translational potential of shuttle peptides for protein and ABE8e-Cas9 RNP delivery to respiratory epithelia in the rhesus monkey model. Following a single aerosol administration, we successfully delivered a fluorescently labelled protein cargo to the epithelial cells of large and small airways, and to some alveolar epithelia. Using the S315 shuttle for ABE8e-Cas9 RNP delivery, we attained significant A to G editing of the *CCR5* locus in cells recovered using bronchial brushing. The editing efficiency of the *CCR5* site in epithelia harvested from the trachea and proximal airways reached 5.3%. Application of this delivery approach in human CF airway epithelia with the *R553X* mutation achieved similar levels of editing and conferred partial restoration of *CFTR* function.

We previously reported the feasibility of CRISPR nuclease RNP delivery to human airway epithelial cells *in vitro* and the airway epithelia of mice *in vivo* using shuttle peptides^12^. The demonstration of successful adenine base editing in rhesus airway epithelia provides further support for translational protein cargo delivery using the versatile shuttle peptide technology. In these studies, the S315 shuttle provided more efficient ABE8e-Cas9 RNP delivery than the S10 shuttle used previously^12^. This likely reflects peptide modifications that included a decreased overall charge density and/or the disruption of cationic charge clusters. Despite a lower GFP delivery activity on CFF-16HBEge cells, these modifications allowed the S315 peptide to maintain its activity in presence of Cas9 RNP (Fig. 1c) which may explain its superiority in Cas9 and ABE8e-Cas9 RNP delivery to well differentiated human airway epithelial and rhesus tracheal epithelial cells *in vitro* (Fig. 2a, b) and to rhesus airway epithelia *in vivo* (Fig. 4c).

To achieve non-CF levels of Cl^−^ transport, it is estimated that *CFTR* function should be restored in 5–50% of the airway epithelial cells^19, 20, 21, 22, 23^. We note that CF patients with mutations associated with as little as 10% residual *CFTR* function may have mild disease phenotypes, including little or no lung disease^24^. Importantly, not all cell types participate equally in Cl^−^ secretion. Several recent scRNA-seq studies have elucidated a diversity of cell types in the large airway surface epithelium (e.g., basal, secretory, goblet, club, ciliated, ionocyte, neuroendocrine, hillock, etc.)^25, 26, 27^. One notable finding from these studies is that *CFTR* transcript abundance varies greatly among individual cell types. Okuda and colleagues used scRNA-seq, single cell RT PCR, and scRNA *in situ* hybridization to demonstrate that secretory cells are the dominant airway surface cell type for *CFTR* expression and function^27^. Ciliated surface cells exhibited low and infrequent *CFTR* expression^27^. While ionocytes express the highest levels of *CFTR* transcripts, they are a rare cell type and their function is still a subject of study. Our results in human airway epithelia with the *R553X* mutation indicate that A to G editing of ~ 5% of surface epithelia partially restored *CFTR* function. This suggests that restoring *CFTR* function in a small proportion of surface epithelial cells may be beneficial. More studies in CF disease models are needed to better understand the percentage of cell types that must be corrected to achieve therapeutic levels of *CFTR* function.

Several factors may influence the outcome of ABE8e-Cas9 RNP delivery including the targeted cell types, the amount of RNP delivered, the gRNA affinity for its target, and the accessibility of the RNP to target DNA. While *CCR5* is not an abundant transcript in airway epithelia^28^, its low-level expression suggests accessible open chromatin. For successful Cas9 RNP delivery, we believe that the quantity of delivered cargo may not be limiting. Rather, the shuttle peptides that achieve delivery to the greatest percentage of cells are expected to exhibit the most efficient editing, independent of the quantity of material delivered.

This study has advantages and limitations. An advantage is the demonstration of the feasibility of this approach in the young rhesus monkey model with airway anatomy and physiology that closely represents humans. We also established that screening of peptides in the air-liquid interface culture model of well-differentiated human airway epithelia successfully identified shuttle peptides with improved *in vivo* delivery properties. The delivery of base editors as RNPs offers advantages as the duration of exposure to the editing agent is short and ends as the protein is degraded. This is expected to limit immunogenicity and off-target editing. Gene editing was achieved without evidence of toxicity as assessed by hematology, clinical chemistry panels, and post-administration monitoring of physical signs^12^.

Limitations of this in vivo primate study include the small sample size for each experimental condition, the administration of a single ABE8e-Cas9 RNP dose, and a single one-week endpoint. Future goals include the evaluation of reagent formulation options and the testing of alternative delivery devices. We suspect that repeated administration of the peptide and cargo would increase the number of edited airway surface cells but could result in immune responses. It is possible that edited surface cell types may persist for long time periods. Our results in Ai9 mice with MAD7 nuclease editing demonstrate the persistence of edited cells *in vivo* over a 1 year period with some decrease at the last time point (Fig. 6). Previous studies in mice demonstrated that ciliated cells of the large and small airways are long-lived (half-life of 6 months in the trachea and 17 months in bronchioles^29^). Detailed information regarding airway cell turnover in humans is unavailable. Our results suggest that redosing of base editing RNPs in the airways could be necessary at intervals of 1 year or greater.

Editing of accessible progenitor cell types is expected to result in permanent correction of the targeted cell and its progeny. There are several regional progenitor cell types in the conducting airways^30^ including basal cells (Muc5AC^−^, K5^+^, p63^+^) in the proximal cartilage containing tracheobronchial epithelium^31, 32^, club cells, and a population of basal and α6β4^+^ cells in the small airways^33, 34^. Of note, there is some evidence that basal cells in the proximal airways have cell membrane extensions that reach the lumen of the airway^35^, while the progenitor cells of the small airways (basal, club, and α6β4^+^ cells) are directly accessible from the lumen^33, 34^. Furthermore, there may be conditions in which secretory cells serve as progenitor cell types^36, 37^.

In summary, a single shuttle-peptide mediated delivery of ABE8e-Cas9 RNPs to the airways of the young rhesus monkey model achieved up to 5.3% editing efficiency in airway epithelia. This finding supports the feasibility of attaining clinically relevant levels of gene editing using this protein delivery technology.

## Methods

Methods, including statements of data availability and any associated accession codes and references, are available in the online version of the paper.

## Data Availability

All relevant data supporting the key findings of this study are available within the article and Supplementary Information files or from the corresponding authors upon reasonable request. The source data for all figures are provided with the paper.
